# Complete eradication of chronic lymphocytic leukemia with unusual skin involvement of high mitotic index after time‐limited venetoclax/obinutuzumab treatment

**DOI:** 10.1002/ccr3.4514

**Published:** 2021-07-23

**Authors:** Maria Dimou, Theodoros Iliakis, Vasileios Paradalis, Aikaterini Bitsani, Marie‐Christine Kyrtsonis, Panayiotis Panayiotidis

**Affiliations:** ^1^ First Department of Internal Medicine, Propaedeutic, Hematology Clinical Trial Unit Laiko General Hospital Athens Greece

**Keywords:** CLL/SLL, MRD negativity, skin nodules, time‐limited treatment, venetoclax

## Abstract

The novel time‐limited combinations with the bcl‐2 inhibitor venetoclax can induce deep responses even in CLL cases with unusual and biologically aggressive presentations, like the skin masses of our patient.

## Graphical abstract

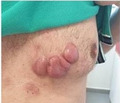

## 1 CASE PRESENTATION

A 51‐year‐old male patient was presented with large skin masses of the chest wall that were pathologically proven to be chronic lymphocytic leukemia/small lymphocytic lymphoma (CLL/SLL) with Ki67:70%. The patient was treated with the time‐limited venetoclax/obinutuzumab combination for 12 cycles and CLL/SLL including the skin component was set in complete remission.

**FIGURE 1:**
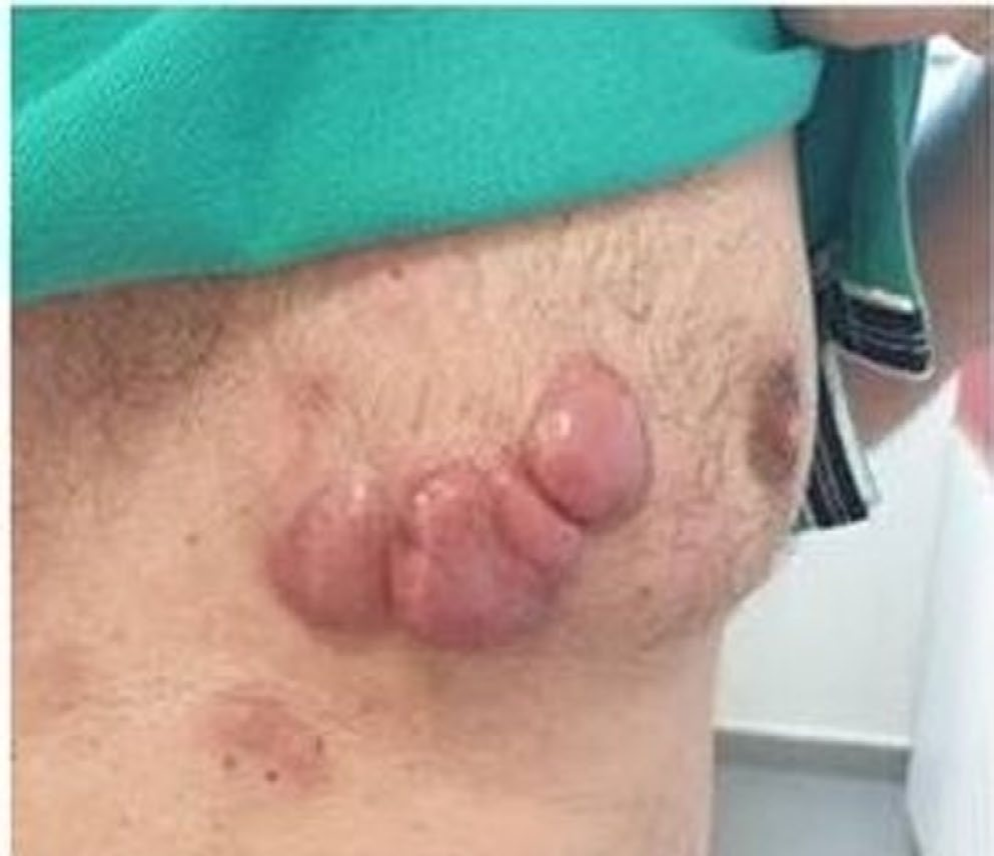
Skin masses at CLL diagnosis

**FIGURE 2:**
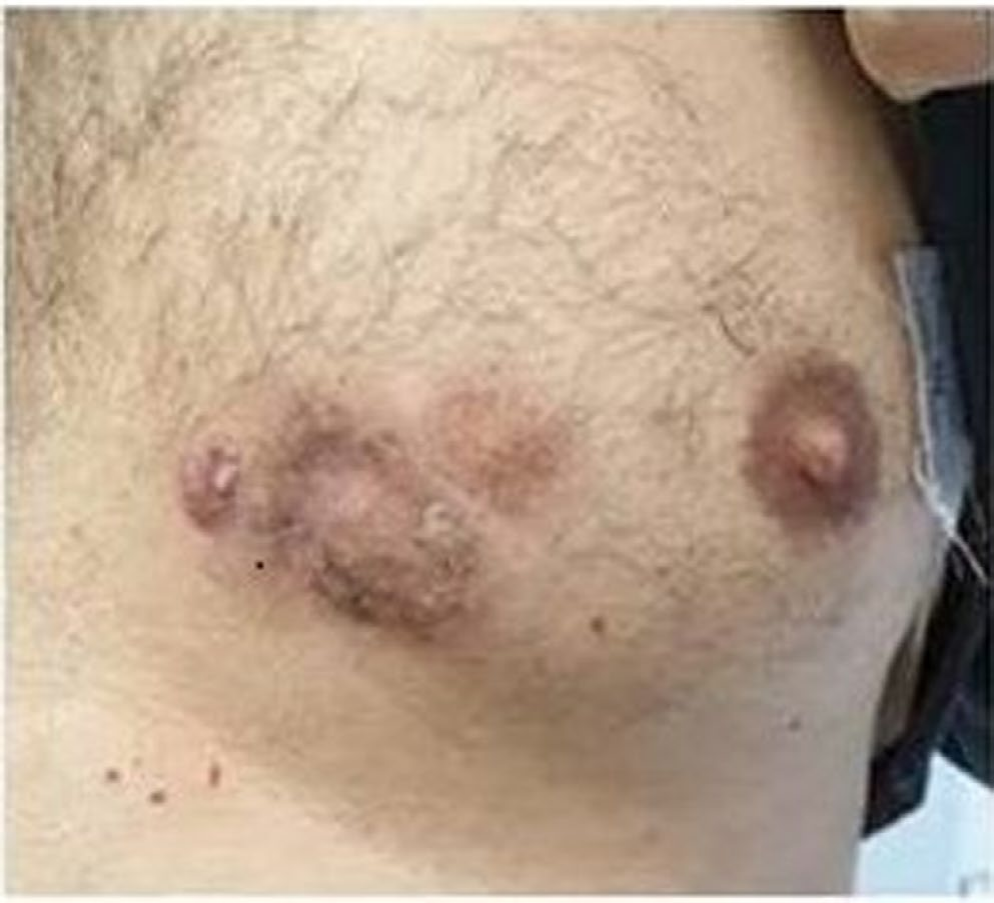
Skin presentation after 6 cycles of venetoclax‐obinutuzumab

**FIGURE 3:**
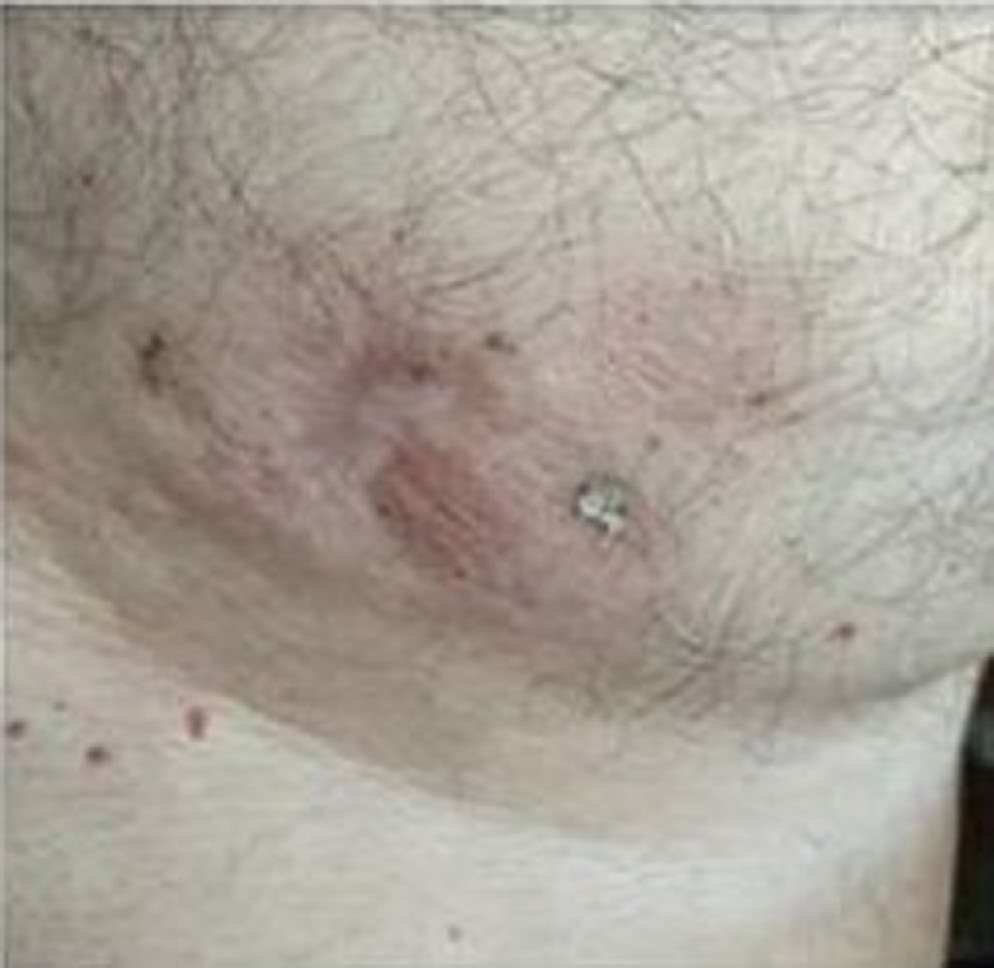
Skin presentation at the end of treatment

A 51‐year‐old male patient was presented with skin masses, 10 × 5 cm (Figure 1), in May 2019. Biopsy showed CLL/SLL: diffuse infiltration by small lymphocytes with the immunophenotype (IF) of CD5+, CD23+, CD79a+>CD20+. Ki67% expression was 70%. Mild lymphocytosis (lymphocytes=13910/mm^3^) and lymphadenopathy without splenomegaly/hepatomegaly in computed tomography (CT) scans were also present. CLL was IgVH unmutated, and deletion 17p/TP53 mutations were negative. The patient was treated with the novel time‐limited combination of venetoclax/obinutuzumab^1^ from July 2019 until July 2020. The CLL skin component responded impressively (Figure 2 and Figure 3). Complete remission in CT scans and bone marrow biopsy was confirmed at the end of treatment. Undetectable CLL minimal residual disease (MRD) in peripheral blood and bone marrow by 8‐color flow cytometry was also confirmed. CLL skin involvement is a rare clinical presentation especially at diagnosis. Ki67>45% is associated with more aggressive biology of Non‐Hodgkin lymphomas.^2^ Our case indicates that the novel time‐limited treatment with the venetoclax /obinutuzumab combination can eradicate the CLL disease, even with unusual and biologically aggressive presentations.

## CONFLICT OF INTEREST

None to declare.

## AUTHOR CONTRIBUTIONS

Maria Dimou MD – author. Theodoros Iliakis MD, co‐author. Vasileios Pardalis MD, co‐author. Aikaterini Bitsani MD, co‐author. Marie‐Christine Kyrtsonis, co‐author. Panayiotis Panayiotidis, co‐author.

## INFORMED CONSENT

Informed consent was obtained from the patient in order to use the images of the skin lesions.

### DATA AVAILABILITY STATEMENT

Data sharing not applicable to this article as no datasets were generated or analysed during the current study.

## References

[1] Fischer K , Othman AS , Fink AM , et al. Venetoclax and Obinutuzumab in Patients with CLL and Coexisting Conditions. N Engl J Med. 2019;380:2225‐2236. 31166681 10.1056/NEJMoa1815281

[2] Broyde A , Boycov O , Strenov Y , Okon E , Shpilberg O , Bairey O . Role and prognostic significance of the Ki‐67 index in non‐Hodgkin's lymphoma. Am J Hematol. 2009;84(6):338‐343. 19384938 10.1002/ajh.21406

